# pH-Responsive mPEG-PLGA/Dexamethasone Coatings for Corrosion Control and Osteo-Immune Modulation of Biodegradable Magnesium

**DOI:** 10.3390/polym18020303

**Published:** 2026-01-22

**Authors:** Yu-Kyoung Kim, Seo-Young Kim, Yong-Seok Jang, Min-Ho Lee

**Affiliations:** Department of Dental Biomaterials, Institute of Biodegradable Materials, School of Dentistry, Jeonbuk National University, Jeonju 54896, Republic of Korea; yk0830@naver.com (Y.-K.K.);

**Keywords:** pH-responsive polymer coating, biodegradable magnesium, mPEG-PLGA, PLGA coating, biodegradable implant

## Abstract

This study aimed to control rapid localized corrosion and inflammation of biodegradable magnesium implants by developing a pH-responsive mPEG-PLGA coating loaded with dexamethasone (Dex). The mPEG-PLGA layer was designed to selectively degrade in alkaline conditions, thereby moderating pH elevation at the implant surface while enabling controlled Dex release. By varying the molecular weight of mPEG and PLGA, the degradation rate and microsphere size were tunable, allowing adjustment of the drug release profile. Among the tested coating solution concentrations (1.5–7.5 mg/mL), the formulation with 3 mg/mL Dex yielded a final cumulative release concentration of 0.02 mg/mL over a two-week period, which suppressed inflammatory responses in RAW 264.7 macrophages with minimal cytotoxicity, while enhancing BMP-2 and RUNX2 expression in mesenchymal stem cells. In a rat femur defect model, Mg implants coated with mPEG-PLGA containing 3 mg/mL Dex significantly increased bone volume and bone mineral density and reduced early TNF-α expression, accompanied by continuous new bone formation and strong BSP-positive osseointegration. These findings suggest that the proposed pH-responsive mPEG-PLGA/Dex coating provides a promising strategy to simultaneously regulate corrosion, attenuate inflammation, and promote bone regeneration around magnesium implants.

## 1. Introduction

Representative biodegradable metallic materials are attractive for bone graft and fixation applications, but their clinical translation is still limited by rapid and localized corrosion [1]. Therefore, controlling the degradation of magnesium alloys in the biological environment remains a challenge with regard to their active application as degradable implants. To tailor the dissolution rate to the intended application and specific implantation site, numerous studies are underway to address this issue through physicochemical surface modifications, such as forming oxide films or polymer coatings, or through alloying [2].

Magnesium metal decomposes via electrochemical ionization, generating Mg^2+^ and OH^−^ at the metal–fluid interface when exposed to body fluids. The resulting hydroxide ions raise the local pH and initially promote passivation by forming a Mg(OH)_2_ surface layer; however, as corrosion progresses and this layer becomes unstable, dissolution accelerates and the entire screw can rapidly degrade [3,4]. Living tissues in a rapidly alkaline environment experience inflammatory reactions or necrosis, which reduces bone cell formation and bioadhesion, leading to implant failure [5]. Acid–base balance plays an important role in influencing the behavior of osteoblasts and, thus, the bone remodeling process [6]. The H_2_ rapidly produced during this process can accumulate around the implant and form gas pockets that induce tissue necrosis [7]. A possible solution to this problem is to use a coating that reduces the rate of Mg decomposition and thus controls the rate of H_2_ formation and alkalinization early in the implantation process. In previous studies, many technologies for coating with biodegradable polymers work by neutralizing the alkalinity of the magnesium corrosion product, as the polymer coating layer becomes acidic upon dissolution. Natural biodegradable polymers such as collagen, polysaccharides such as chitosan, and synthetic biodegradable polymers such as polycaprolactone (PCL) and poly(lactic-co-glycolic acid) (PLGA) demonstrate excellent protection and biocompatibility on magnesium [8,9]. Contrary to theoretical principles, biodegradable polymer coatings partially delaminate and hydrolyze, lowering the surrounding pH, which leads to persistent and localized corrosion of magnesium [8]. However, most PLGA-based coatings reported to date have been designed simply as passive barriers, without coupling their degradation behavior to the corrosion-driven pH changes at the Mg interface or integrating an optimized local drug-delivery function. In particular, there is a lack of systems in which the PLGA matrix is engineered to dissolve preferentially under alkaline conditions generated by Mg corrosion, while simultaneously releasing an anti-inflammatory and osteogenic agent at a concentration window that is biologically effective yet non-toxic.

Therefore, in this study, we designed a mPEG-PLGA-based coating that is not only biodegradable but also functionally coupled to the Mg corrosion microenvironment, selectively dissolving in the alkaline domain that forms during corrosion while remaining stable under physiological pH. By combining this pH-responsive PLGA matrix with a finely tuned, suitable dexamethasone payload, our approach aims to go beyond conventional PLGA barrier coatings and establish a corrosion-coupled, locally immunomodulatory system for Mg implants.

Polymers that dissolve in alkaline solutions include polyacrylic acid (PAA), polyvinyl alcohol (PVA) [10], poly(L-lactide) [11], and polylactic acid-co-glycolic acid (PLGA). PLGA is a copolymer of polylactide (PLA) and polyglycolide, and is a biodegradable polymer that can be naturally converted into non-toxic degradation products over time in the body [12]. Polyethylene glycol (PEG), a hydrophilic polymer widely recognized for its excellent biocompatibility, also possesses the notable capacity to form coordination complexes with various metal ions [13]. mPEG-PLGA was used in this study on the assumption that it is selectively soluble in alkaline solutions, primarily due to accelerated hydrolysis of ester bonds [14]. When local corrosion elevates pH at the Mg–tissue interface, rapid dissolution of the mPEG-PLGA layer is expected to (i) buffer the alkaline shift and (ii) dynamically modulate exposure of the Mg substrate. We used mPEG-PLGA in which the lactide–glycolide ratio was kept constant at 50:50, while the molecular weight of the PLGA block was systematically varied from Mn 10,000 to 30,000 to adjust dissolution rate and microsphere formation [15].

Additionally, this study included dexamethasone (Dex) within the coating layer to effectively control the initial inflammatory response and promote bone formation. Dexamethasone is a synthetic glucocorticoid with potent anti-inflammatory and immunomodulatory properties [16]. Dexamethasone helps promote osteoblast differentiation and bone formation, and thus, it has the potential to simultaneously promote bone regeneration while controlling inflammation through sustained drug release from the coating on the magnesium surface. High concentrations of dexamethasone can induce osteoblast death or inhibit differentiation, and chronic overdose can cause common side effects of glucocorticoids (osteoporosis, muscle weakness, etc.) [17]. Therefore, the purpose of this study was to optimize the drug dosage required for effective bone formation and inflammation control, and to suppress local corrosion and inflammatory reactions around magnesium implants by using a pH-responsive mPEG-PLGA coating combined with a dexamethasone sustained-release system, thereby improving the biocompatibility and bone regeneration capacity of the implants.

## 2. Materials and Methods

### 2.1. Synthesis of Materials and Surface Treatments

In this study, mPEG-PLGA (BroadPharm, San Diego, CA, USA) was used with a fixed lactide–glycolide ratio of 50:50 and a PEG block of average Mn 5000, while the PLGA block molecular weight was varied between Mn 10,000 and 30,000.

Coating layers on metal surfaces are subject to variables such as adhesion and mechanical peeling, making it difficult to quantitatively assess how the polymer itself degrades and dissolves in neutral and alkaline environments. Therefore, microparticles were fabricated as a model system to evaluate the unique pH-responsive degradation behavior of mPEG-PLGA and optimize the PLGA molecular weight prior to Mg substrate coating. mPEG-PLGA microparticles were first prepared in polyvinyl alcohol (PVA) (Mw 8000–10,000, 360627, Sigma Chemicals, St. Louis, MO, USA) using a solid-in-oil-in-water solvent evaporation method to evaluate the pH-responsive degradation behavior.

Briefly, mPEG-PLGA was dissolved in methylene chloride to prepare a 5 wt% polymer solution (5 mL). This organic phase was added dropwise into 100 mL of a 2% (*w*/*v*) PVA solution at a rate of 50 μL/s under magnetic stirring at 800 rpm. The mixture was pre-emulsified at room temperature for 5 min. Subsequently, the emulsion was stirred at 500 rpm for 3–4 h to ensure complete evaporation of the methylene chloride. The resulting hardened microspheres were recovered by centrifugation (5000× *g* for 5 min), washed five times with distilled water to remove residual PVA. The washed microspheres were then frozen at −80 °C for at least 4 h and subsequently lyophilized for 24 h to obtain dry microparticles.

The shape of the fine particles was observed using SEM, and the specimens were immersed in simulated body fluid (SBF; Hank’s balanced salt solution, H2387, Sigma, pH 7), and in an alkaline solution prepared by dissolving pure magnesium in SBF at 37 °C for 72 h to make a pH of 10 and then incubated for up to 5 days according to ISO 10993-12 [18]. The degradation state was measured by absorption spectroscopy using a UV–vis (V-630; JASCO, Tokyo, Japan) and evaluated by a scanning electron microscope (SEM) (JSM-6400, JEOL, Tokyo, Japan).

Pure magnesium (Mg 99.9%, Goodfellow, Huntingdon, Cambridgeshire, UK) plates (5 × 10 × 2 mm) were used as substrates. Mg samples were dip-coated by immersing them in a 5 wt% mPEG-PLGA (Mn 10,000–30,000) solution in dichloromethane (Showa Chemical, Minato City, Japan) and withdrawing at 20 mm/min, followed by drying at 25 °C for 10 min. Dexamethasone (D4902, Sigma Chemicals, St. Louis, MO, USA) was dispersed in the coating solution at concentrations of 1.5~7.5 mg/mL. Commercially pure Ti (99.9% purity, Kobe Steel, Kobe, Japan) of the same size was used as a comparative control substrate. The dip-coating procedure was repeated for four cycles by a dip coater (Micro Dip Coater EF-5100, E-Flex, Bucheon, Republic of Korea). The samples were dried at 37 °C for 24 h in a drying oven to remove residual organic solvent and moisture.

### 2.2. Surface Characterization

The mPEG-PLGA raw material, mPEG-PLGA microparticles, and mPEG-PLGA coating on Mg were analyzed to confirm the presence of polymer synthesis and its chemical composition using Fourier-transform infrared (FT-IR) spectroscopy (Spectrum GX, PerkinElmer, Waltham, MA, USA) at 500–4000 cm^−1^. The surface morphologies during various treatments were observed by SEM. The surface roughness was detected by atomic force microscopy (AFM; Innova, Bruker, Karlsruhe, Germany) using a contact mode with a scan area of 5 × 5 μm^2^ under N_2_ gas. Each sample was scanned 7 times, and the root mean square (RMS) surface roughness (Rq) was calculated. Contact angles were measured as the sessile-drop type by a contact-angle analyzer (Phoenix-300 Touch, Surface Electro Optics, Suwon, Republic of Korea) for wetting energy and work of adhesion value.

### 2.3. Immersion Test

The pH changes in the medium was monitored for 60 h in SBF, and sample weight change was measured over 7 days and expressed as a percentage of the initial sample weight (W_final_/W_initial_ × 100%). The standard calibration curve was prepared by dissolving dexamethasone in distilled water (DW) at four concentration points ranging from 0.025 to 0.1 mg/mL, with three replicates per concentration. Distilled water was selected as the solvent to eliminate potential interference from inorganic ions (e.g., Ca^2+^, PO_4_^3−^, HCO_3_^−^) that could contribute to background absorbance and compromise the accuracy of drug quantification. The linear regression of the calibration curve yielded excellent linearity (R^2^ > 0.99) with a slope (m) of 25.14857 and y-intercept (b) of 0.12119.

At each time point during the release study, the absorbance of the release medium was measured at λ_max = 242 nm. The dexamethasone concentration at each time point (C_t_, mg/mL) was calculated using the calibration curve parameters:

C_t_ = (A_t_ − 0.12119)/25.14857

where A_t_ is the measured absorbance at time t. The cumulative dexamethasone release at each time point was then recorded and plotted against time (h or days) to generate the release profile. All absorbance measurements were performed in triplicate (*n* = 3 samples per time point), and results are expressed as mean ± standard deviation. Representative UV–Vis spectra of the standard dexamethasone solutions and release medium at key time points (6 h, 1 day, 7 days, and 21 days) are provided in Supplementary Figure S1.

The quantitative analysis of Mg, Ca in the extract solution (according to ISO 10993-12, sample size 1.25 cm^2^/mL in the SBF, 36 °C, eluted for 48 h) was performed 7 times by using Inductively Coupled Plasma Mass Spectrometry (ICP-MS, Agilent 7500a, Agilent Technologies, Santa Clara, CA, USA). Statistical significance was analyzed by Mann–Whitney U Test (*p* < 0.05 considered significant [*]; *p* ≥ 0.05 considered not significant [#]).

### 2.4. Inflammatory Cytokines in Macrophage RAW 264.7 Macrophages

RAW 264.7 cells from KCTC (KCLB 40071, Seoul, Republic of Korea) were grown in Dulbecco’s modified Eagle’s medium (DMEM, Gibco, Grand Island, NY, USA) supplemented with 10% fetal bovine serum (FBS; Gibco, USA). Cells were plated at 2 × 10^5^ cells/mL in 24-well culture plates and cultured for 6 h. After the removal of the media, D-MEM (with 0.5% FBS) was exchanged and cultured for 18 h. Extract solutions were prepared by incubating the coated Mg samples in DMEM with 0.5% FBS according to ISO 10993-12 and then supplemented with 0.5 μg/mL lipopolysaccharide (LPS). The control groups were D-MEM with 0.5% FBS, DMEM with 10% FBS, and DMEM with 0.5% FBS + 0.5 µg/mL LPS. Tumor necrosis factor-alpha (TNF-α) and Interleukin-1 beta (IL-1β) were measured using the TNF-α and IL-1β ELISA kit (BMS607-3 and BMS6002, Thermo fisher, Waltham, MA, USA) by reading absorbance at 405 nm.

### 2.5. Osteoblast Cell Viability

MC3T3-E1 pre-osteoblasts (ATCC, Manassas, VA, USA) were seeded at a density of 2.5 × 10^4^ cells per well in 24-well plates. After incubation for 2 and 4 days with the sample extracts, cell viability was assessed using a standard water-soluble tetrazolium salt (WST-8) assay kit (96992, Sigma, St. Louis, MO, USA). And the product quantity of H_2_O_2_ from extract solution of samples was analyzed using the Biovision hydrogen peroxide assay kit (Biovision Research Products, Milpitas, CA, USA) after incubation for 2, 4 and 10 days. The H_2_O_2_ content in samples was determined by comparison with a standard curve. To evaluate osteoblastic and osteoclastic differentiation, the cultured cell for 14 days was washed with 0.9 wt% NaCl, and extraction solution (saline including 1% NP-40) was added to solubilize adherent cells to each well. For ALP activity, 0.2 M Tris–HCl buffer containing p-nitrophenyl phosphate (pNPP) was added to each well and incubated at 37 °C for 60 min using an ALP assay kit (MK301, Takara, Shiga, Japan), and substrate solution as 0.5 M sodium acetate buffer and 0.5 M sodium tartrate with p-nitro-phenyl phosphate (pNPP) was added to react for TRACP assay. The absorbance was measured at 405 nm using a microplate spectrophotometer (EMax, Molecular Devices, San Jose, CA, USA).

### 2.6. The Expression Proteins Were Detected by Western Blot

Rat bone-marrow-derived mesenchymal stem cells (BMSCs) were isolated from the femur and tibia bone marrow of 8-week-old male Sprague Dawley rats (270–280 g, Damul Science, Daejeon, Republic of Korea). All procedures using animal tissues were approved by the Institutional Animal Care and Use Committee of Jeonbuk National University, Republic of Korea (NON2024-091). The cells were seeded in low-glucose Dulbecco’s modified Eagle’s medium (DMEM, Gibco, Grand Island, NY, USA) supplemented with 10% fetal bovine serum (FBS; Gibco, Grand Island, NY, USA) and GlutaMax^®^ (Thermo Fisher Scientific Inc.) at 37 °C in humidified air with 5% CO_2_. The 3–5-passage cells were cultured at a density of 2 × 10^5^ cells/24 wells. Protein was extracted from the BMSCs using a lysis buffer containing 150 mM NaCl, 5 mM EDTA, 50 mM Tri-HCl (pH 8.0), 1%-NP 40, 1 mM aprotinin, 0.1 mM leupeptin, 1 mM pepstatin, and quantified using the Bradford dye-binding assay (Bio-Rad, Hercules, CA, USA). Immunoblot analysis was performed as previously described [19]. Briefly, protein samples were separated by 8–15% sodium dodecyl sulphate–polyacrylamide gel electrophoresis under denaturing condition and electroblotted onto nitrocellulose membranes. Membranes were incubated with a specific primary antibody (1:1000 to 1:2000 dilution) for 24 h at 4 °C, followed by incubation with a horseradish peroxidase-conjugated secondary antibody for 1 h at room temperature. Signals were visualized using LAS-4000 CCD imaging system (Fujifilm, Tokyo, Japan) after applying chemiluminescence detection reagent (Amersham Pharmacia Biotech, London, UK) according to the manufacturer’s protocol. Protein expression levels were analyzed using ImageQuant TL 8.1 software (GE Healthcare Bio-Science, Uppsala, Sweden). Antibodies against bone morphogenetic protein (BMP)-2 (BS3473), BMP-7 (BS3674), osteoprotegerin (OPG, BS1862), osteopontin (OPN, BS1264), and β-actin (AP0060) were purchased from Bioworld (Minneapolis, MN, USA). Antibodies against p-ERK (#4370) were purchased from Cell Signaling Technology (Danvers, MA, USA). The Western blot band intensities were measured with ImageJ software (US National Institutes of Health, Bethesda, MD, USA). Statistical analysis of Western blot data was performed using the Mann–Whitney U test (SPSS ver. 12.0 K, Chicago, IL, USA), with *p* < 0.05 considered statistically significant.

### 2.7. In Vivo Evaluation of Bone Regeneration by Adding an Implant into Rat Tibia

12 male Sprague Dawley rats (270–280 g, Damul Science, Daejeon, Republic of Korea) were used for in vivo, and this was approved by the Institutional animal ethics committee of Jeonbuk National University, Republic of Korea (IRB: NON2025-106-001). Anesthesia was induced by intramuscular injection of zoletil (0.5 mL/100 g; Virbac, Carros, France) and xylazine hydrochloride (0.1 mL/100 g; Rompun, Bayer Vital, Leverkusen, Germany). A 2 mm diameter hand piece (X-smart endodontic motor, Dentsply Maillefer, Ballaigues, Switzerland) was used to make a hole with a depth of 7 mm in both sides of femur. Four screws per group were implanted into these defects by hand insertion. Six rats were sacrificed at 3 weeks and the remaining six at 6 weeks. The femur sections with the implants were cut and fixed in 10% neutral-buffered formalin. Dynamic bone histomorphometry was performed using sequential double-fluorochrome labeling around the implants. The first label (red) was 15 mg/mL alizarin complexone in 12.5 g/L NaHCO_3_ injected at 1.67 mL/kg on the day of surgery (3-week group) or at week 2 (6-week group). The second label (green) was 20 mg/mL calcein in 12.5 g/L NaHCO_3_, injected at 1.25 mL/kg 2 weeks after the first label in each group. The specimens were decalcified in 15% EDTA for 6 weeks and embedded in paraffin. Paraffin-embedded specimens were sectioned at 6 μm using a microtome (RM2255, Hamburg, Germany). Masson’s trichrome staining was performed by Stain Kit (VitroVivo Biotech, Rockville, MD, USA) and the specimens were examined under an optical microscope to determine new bone formation and bone maturation. Immunohistochemical staining of Mouse TNF-alpha antibody (52B83) (NB600-1422, Novus Biologicals, Centennial, CO, USA), and bone sialoprotein (BSP) (SC-73497; Santa Cruz Biotech., Inc., Dallas, TX, USA) served to identify local inflammatory response and newly formed, mineralizing bone matrix at the bone–implant interface by using optical microscopy (DM 2500 M, Leica, Wetzlar, Germany). Scanning by micro-CT (SKYSCAN 1076, Skyscan, Kontich, Belgium) occurred at 3 and 6 weeks to determine the degradation of the screw. The dataset images were reconstructed by the Bruker-Skyscan free software (^®^DataViewer, ^®^CTAn, and ^®^CTvox, Kontich, Belgium). The volume (mm^3^) of the implant after implantation was calculated using the 3D analysis plugin in the CTAn.

## 3. Results

**Figure 1:**
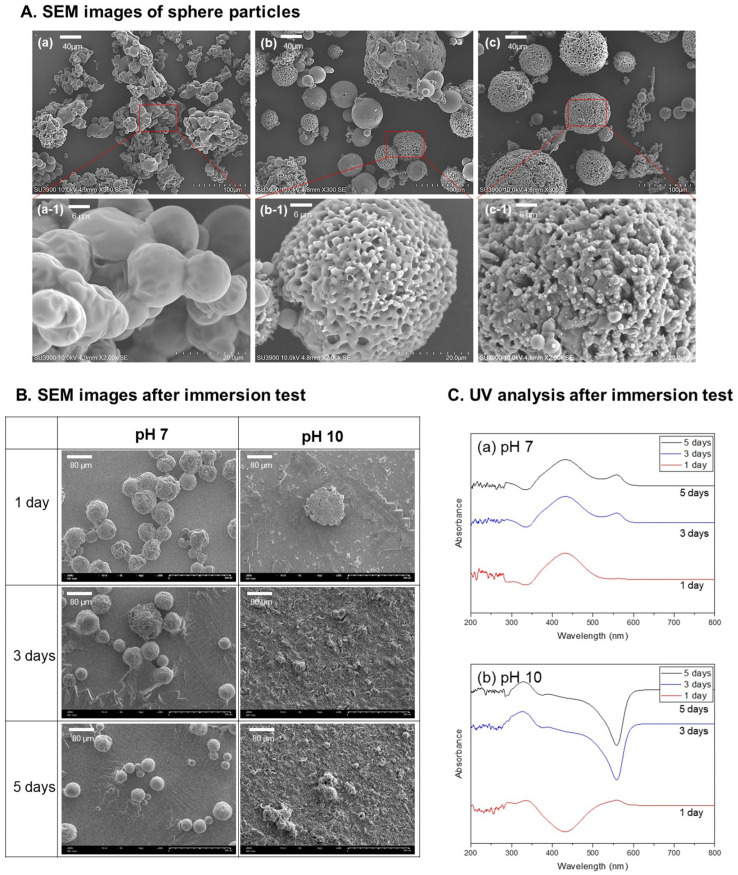
Morphology and pH-dependent degradation of mPEG-PLGA spheres. (**A**) SEM images of mPEG-PLGA spheres with PLGA Mn 10,000 (**a**), 20,000 (**b**), and 30,000 (**c**), and higher-magnification views (**a-1**,**b-1**,**c-1**). (**B**) SEM images and (**C**) UV–vis spectra of Mn 20,000 spheres after 5-day immersion at pH 7 and pH 10.

To establish a magnesium surface treatment method using a pH-responsive polymer, the selection of the polymer type, the ability to form a coating on the metal surface, and the selective dissolution characteristics in an alkaline environment are key design variables. In Figure 1A, mPEG-PLGA with a fixed lactide–glycolide ratio, and the ability to form stable spherical particles was assessed by systematically varying the molecular weight of the PLGA block. At a low molecular weight (Mn 10,000), spherical particles were formed, but aggregation between particles was prominent and the surface was relatively smooth. When the molecular weight was 20,000 (Figure 1A(b)), single spherical particles were formed, with a mixture of smooth and porous surfaces, and a diameter of approximately 30–60 µm. As the molecular weight increased (Mn 30,000), uniform spheres (diameter of approximately 60–80 µm) with a porous surface were obtained. After selecting mPEG-PLGA spheres with a molecular weight of 20,000 as the representative condition, immersion experiments were performed at pH 7 and pH 10 (Figure 1B). At pH 7, the spherical shape and coating layer were well maintained for 5 days, effectively inhibiting magnesium corrosion. However, at pH 10, the spherical shape collapsed from the first day, and after the third day, a significant portion of the coating layer was dissolved, exposing the magnesium surface. These findings indicate that mPEG-PLGA acts as a protective layer under neutral conditions and, upon corrosion-induced alkalization, selectively dissolves to expose the underlying Mg surface.

As a result of analyzing the eluted solution using a UV–vis spectrophotometer (Figure 1C), the pH 7 group (a) exhibited a cleared absorption peak near 430 nm, and because the polymer spheres were few degraded, the amount of low-molecular-weight degradation products in the solution remained small, resulting in almost identical spectral profiles over time. As immersion time increased, a broad new feature gradually emerged around 557 nm, which is attributed to weak light scattering from a limited number of finely dispersed degradation fragments. Thus, at pH 7 (neutral), polymer decomposition proceeds much more slowly than under alkaline conditions, but gradual structural changes and partial degradation of mPEG-PLGA are expected during long-term exposure.

Under alkaline conditions (b), peaks appeared at wavelengths similar to those in (a), but the overall spectra showed an apparent inversion (negative shift) of the baseline. On day 1 at pH 10, only a modest amount of degradation product was released, so the 430 nm region still primarily reflected absorption from dissolved species and scattering remained relatively minor. However, after 3 and 5 days, extensive alkaline degradation of the spheres generated numerous suspended polymer fragments and fine particles, and particle-induced light scattering became the dominant optical event. As a result, the signal in the short-wavelength region (~330 nm) intensified, the distinct absorption band around 430 nm was masked and effectively disappeared, and the feature near 557 nm evolved into a broad scattering peak accompanied by an apparent negative baseline shift, rather than a simple increase in absorbance.

**Figure 2:**
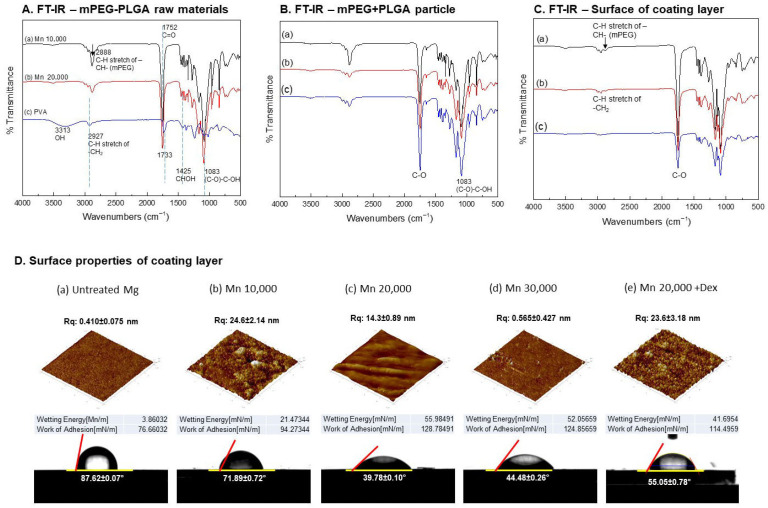
Chemical characterization and surface properties of mPEG-PLGA. (**A**) FT-IR spectra of raw materials: (**a**) Mn 10,000 mPEG-PLGA, (**b**) Mn 20,000 mPEG-PLGA, (**c**) PVA. (**B**) mPEG-PLGA particles and (**C**) FT-IR of coating surfaces: (**a**) Mn 10,000, (**b**) Mn 20,000, (**c**) Mn 30,000. (**D**) AFM and contact angle of coatings: (**a**) untreated Mg, (**b**) Mn 10,000, (**c**) Mn 20,000, (**d**) Mn 30,000, (**e**) Mn 20,000 + Dex.

Figure 2A–C show the chemical structures of mPEG-PLGA raw material, microspheres, and surface-treated mPEG-PLGA, analyzed by FT-IR. In Figure 2A, the raw mPEG-PLGA showed decreasing band intensities at 2888 cm^−1^ (C–H) and 1752 cm^−1^ (C=O) with increasing molecular weight, while the control group, PVA, exhibited a strong O–H signal at 3313 cm^−1^.

Figure 2B shows the synthesized polymer spheres, in which the 1100 cm^−1^ C–O peak gradually increased with increasing molecular weight. Figure 2C shows a strong 1100 cm^−1^ C–O peak intensity on the surface of the coating layer, which decreased with increasing molecular weight. Figure 2D shows the roughness and surface structure of the surface-treated magnesium. At Mn 10,000 (Figure 2D(b)), the roughness increased rapidly but showed an irregular pattern, while at Mn 20,000 (Figure 2D(c)), the roughness decreased and more regular micropatterns were formed. At Mn 30,000 (Figure 2D(d)), the surface returned to a relatively smooth surface similar to the untreated group. When dexamethasone was added, the surface roughness increased overall. The Mn 20,000 coating showed the lowest contact angle (approximately 39.8°), indicating the maximum hydrophilicity, and when Dex was added under the same conditions, the contact angle increased to approximately 55.1°, changing the surface to a more hydrophobic surface.

**Figure 3:**
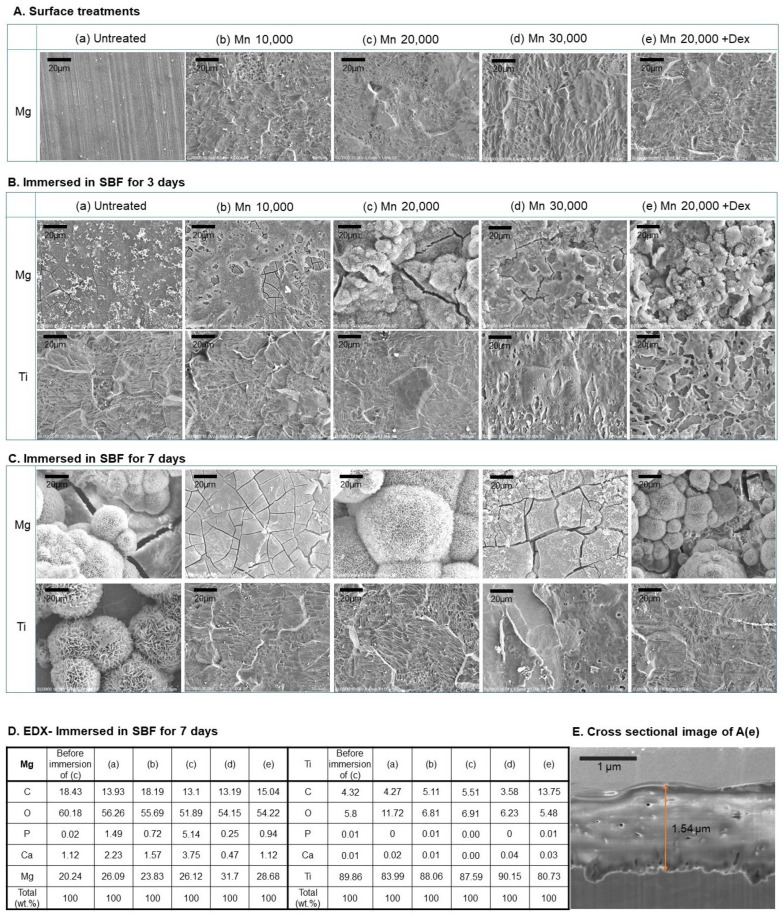
Surface morphology and composition of Mg and Ti after coating and SBF immersion. (**A**) SEM images of Mg: (**a**) untreated, (**b**) Mn 10,000, (**c**) Mn 20,000, (**d**) Mn 30,000, (**e**) Mn 20,000 + Dex. (**B**) SEM images of Mg (**top**) and Ti (**bottom**) after 3-day SBF immersion. (**C**) SEM images after 7-day SBF immersion. (**D**) Elemental composition (wt%) and EDX mapping after 7 days. (**E**) FIB cross-section of the Mn 20,000 + Dex coating.

Figure 3 shows the surface changes observed after mPEG-PLGA treatment on Mg and Ti, respectively, and immersion in body fluids. Both Mg and Ti exhibited uniform coating layer distribution regardless of the difference in mPEG-PLGA molecular weight (Figure 3A). The addition of dexamethasone revealed an uneven particle-like structure on the surface. The film thickness was similar in all surface treatment groups, approximately 1.5–1.7 μm, and a representative image is shown in Figure 3E. After immersion in SBF for 3 days, overall fine precipitation and corrosion traces were observed on the untreated Mg (Figure 3B(a)), whereas in the mPEG-PLGA-coated group, as the PLGA molecular weight increased, the surface tended to be covered with denser spherical precipitates (presumed to be apatite) and a homogeneous pattern. In the Mn 20,000 + Dex group, particles and spherical precipitates were clearly distributed across the surface, with more pronounced agglomeration. On the Ti substrate, overall precipitation formation and surface changes were less visible than on the Mg substrate, and only fine irregularities and grains were observed, and the precipitate layer was thin and uneven. In the Dex group, the increase in Ca and P was relatively low, but spherical deposits similar to the Mn 20,000 coating were partially observed. After 7 days of SBF immersion (Figure 3C), typical spherical and aggregated precipitates of Mg were clearly covering the entire surface of each coating. In particular, many large spherical structures were formed on the Mn 20,000 and Mn 20,000 + Dex, and the precipitate layer was the thickest and most uniform. On untreated Ti, SBF immersion induced the formation of apatite-like precipitates, indicating that the untreated Ti surface was bioactive. In contrast, on Ti substrates coated with mPEG-PLGA, the development of fine spherical apatite structures was almost non-existent, and the original irregular surface pattern of the coating was mainly observed. EDX results (Figure 3D) show that the P and Ca contents are highest in the Mn 20,000 coating (P: 5.14%, Ca: 3.75% based on the Mg surface). This result is consistent with the uniform and thick spherical precipitation (such as apatite) observed in SEM.

**Figure 4:**
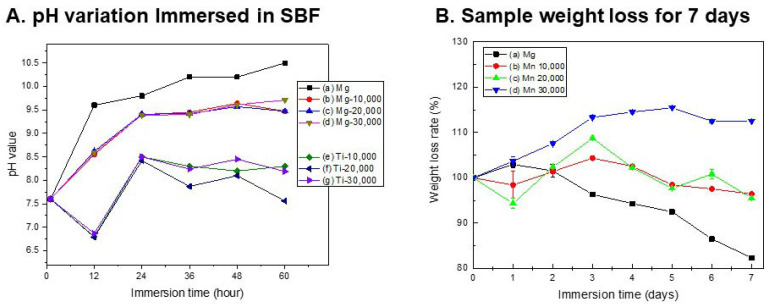
Biological corrosion response analysis as (**A**) pH variation immersed in SBF, and (**B**) Sample weight loss for 7 days.

Figure 4 shows the biodegradability of the coating layer, the pH change in SBF, and the weight loss rate over 7 days. After mPEG-PLGA coating, some of the polymer dissolved over the course of 12 h. In the case of Ti, the pH of the SBF temporarily decreased, but remained below 8.5, indicating a neutral state. In the case of magnesium, a lower pH increase was observed in the first 12 h compared to the untreated group (a) due to the effect of the polymer coating. As the polymer dissolved, the pH of the SBF decreased, and thus, the pH tended to be maintained at 9.2–9.5. In the untreated group, sample weight increased slightly on day 1 and then progressively decreased as corrosion accelerated. All coating groups exhibited superior corrosion resistance compared to the untreated group, and, in particular, the Mn 30,000 coating (Figure 4B(d)) actually showed an increase in mass due to the growth of a dense oxide film and coating layer.

**Figure 5:**
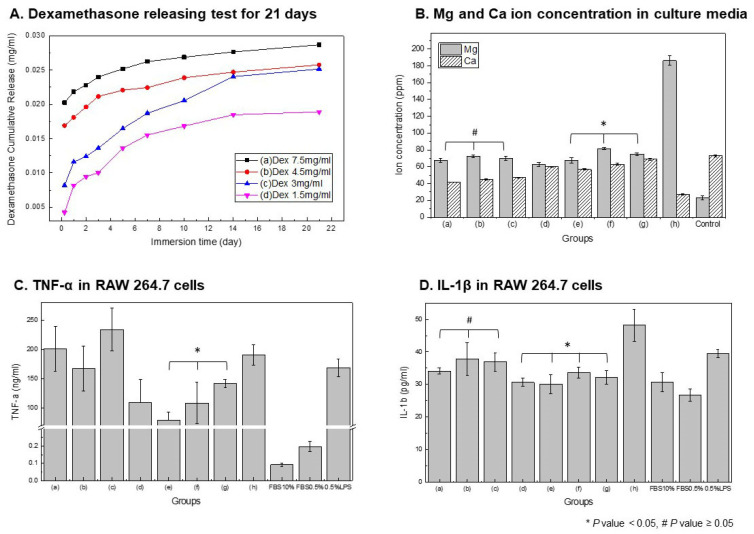
Dexamethasone release kinetics and biological responses of polymer-coated Mg. (**A**) Cumulative Dex release over 21 days. (**B**) Mg and Ca ion concentrations in SBF (ICP). (**C**) TNF-α and (**D**) IL-1β levels in RAW 264.7 cells under identical extract conditions. Sample groups: (**a**) Mn 10,000, (**b**) Mn 20,000, (**c**) Mn 30,000, (**d**) Mn 20,000 + Dex 7.5 mg/mL, (**e**) Dex 4.5 mg/mL, (**f**) Dex 3 mg/mL, (**g**) Dex 1.5 mg/mL, (**h**) untreated Mg, and controls (10% FBS, 0.5% FBS, 0.5% FBS + LPS).

Next, the effect of dexamethasone addition on the polymer coating and cytotoxicity was evaluated. As shown in Figure 5A, the cumulative release of dexamethasone from mPEG-PLGA-coated samples gradually increased in proportion to the administered concentration. The Dex 7.5 mg/mL group (a) showed the highest release rate and cumulative amount, whereas the Dex 1.5 mg/mL group (h) exhibited the slowest and lowest release profile.

Figure 5B shows the results of measuring the Mg and Ca ion concentrations in the culture medium. The ion release patterns allow for an indirect evaluation of the corrosion inhibition and cytotoxic potential of the coating. The mPEG-PLGA-coated samples (a–c) show a tendency for Mg release to decrease, with the effect becoming more pronounced as the mPEG-PLGA concentration increases. Untreated Mg (h) exhibits the highest Mg ion concentration, while the PLGA-coated samples (a–c) show a significant decrease in Mg release, but a decrease in Ca compared to the control group. As shown in the surface edx results in Figure 3D, this is due to Ca adsorption along with oxides on the sample surface. The Dex-added group (d–g) showed a pattern in which the initial Mg ion concentration slightly increased and then decreased over time, while the Ca ion concentration was similar to that of the control group. This suggests that the coating inhibits corrosion while reducing the Ca loss required for bone formation.

Figure 5C,D present analyses of the expression of inflammation in macrophages. Untreated Mg (h) and the coating groups (a–c) induce a strong inflammatory response with high TNF-α secretion. In the Dex-added groups (d–g), TNF-α is significantly reduced compared to the positive control (LPS) depending on the concentration, and Dex 7.5 mg/mL (d) shows the best anti-inflammatory effect (* significant difference confirmed). The secretion of the inflammatory cytokine IL-1β is highest in untreated Mg (h) and tends to be suppressed in the coating groups (a–c). In the Dex-coated groups (d–g), IL-1β secretion gradually decreases with increasing concentration, and, in particular, Dex 7.5 mg/mL (d) is the most effective (* significant difference). The control conditions provided clearly defined pro-inflammatory and baseline reference states against which the experimental groups could be compared.

**Figure 6:**
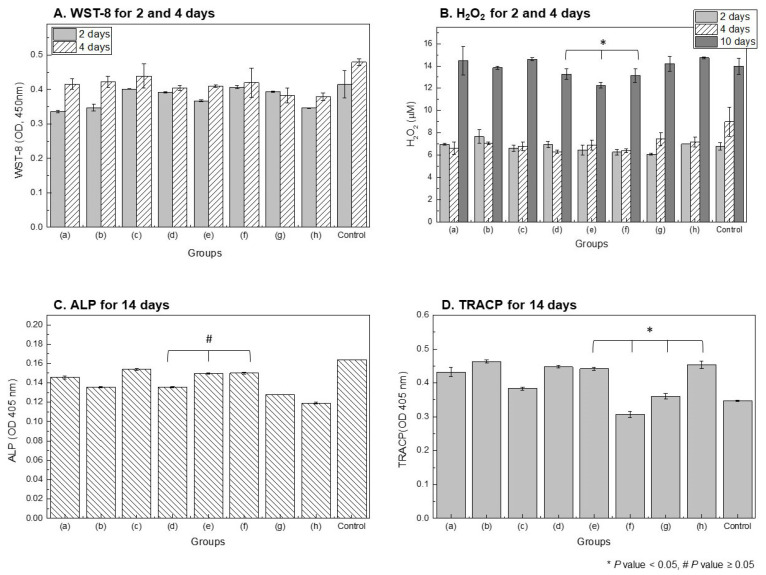
Cytotoxicity and osteoblastic activity of polymer-coated Mg. (**A**) Cell viability (WST-8). (**B**) H_2_O_2_ production in cell culture. (**C**) ALP activity, (**D**) TRACP activity. Sample groups: (**a**) Mn 10,000, (**b**) Mn 20,000, (**c**) Mn 30,000, (**d**) Mn 20,000 + Dex 7.5 mg/mL, (**e**) Dex 4.5 mg/mL, (**f**) Dex 3 mg/mL, (**g**) Dex 1.5 mg/mL, (**h**) untreated Mg, and Control (cells without sample exposure).

WST-8 results for evaluating osteoblast proliferation (Figure 6A) showed that cell viability increased after 4 days compared to 2 days in all experimental groups, with the Dex-coated group (e–g) demonstrating higher viability compared to the untreated group (h) and the mPEG-PLGA-coated group (a–c). However, compared with the negative control, all extract-treated groups showed reduced proliferation, and the Dex-treated groups in particular exhibited minimal additional growth after 4 days. No significant differences were observed among dexamethasone concentrations. H_2_O_2_, an indicator of oxidative stress, was significantly reduced in both the dexamethasone-coated group (e–g) and the control group after 4 days compared to the control group, and the dexamethasone-coated group showed a lower value than the other surface treatment groups after 10 days.

High H_2_O_2_ levels were maintained in the untreated Mg (h) and mPEG-PLGA-coated groups (a–c) (*, significant difference). This suggests that Dex release mitigates reactive oxygen species generation and oxidative stress associated with Mg corrosion.

ALP (alkaline phosphatase, a marker of bone differentiation) in Figure 6C significantly increased in the Dex 4.5–3 mg/mL (e, f) coating group (#, *p*-value not significant), indicating a positive effect on osteoinduction and osteocyte activation. In Figure 6D, TRACP (a marker of bone destruction and osteoclasts) was highest in the untreated group (h) and significantly lower in the low-concentration Dex-coated group (Dex 3–1.5 mg/mL) (*, significant difference). Dex-coated groups above a certain concentration were ineffective in inhibiting bone destruction.

**Figure 7:**
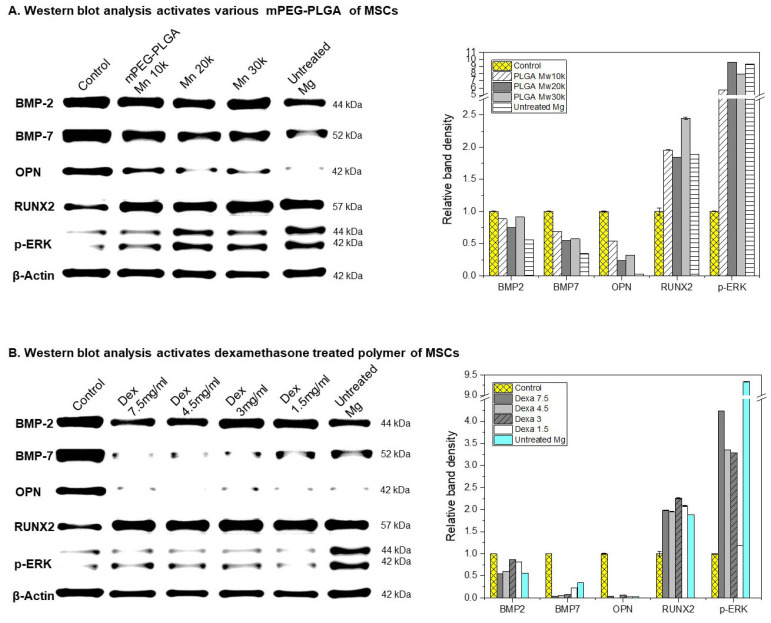
Western blot analysis of osteogenic differentiation markers in MSCs treated with mPEG-PLGA coating layer (**A**), and mPEG-PLGA coating layer with dexamethasone as (**B**), (Control: normal cells).

Figure 7A shows the results of analyzing bone-related protein expression in MSCs (mesenchymal stem cells) in mPEG-PLGA-coated magnesium (Mg) samples. The expression of BMP-2 and BMP-7 in untreated Mg was significantly lower than in the coated and control groups, suggesting a lack of stimulation of osteogenic differentiation. On the other hand, the expression of BMP-2 and BMP-7 in the mPEG-PLGA coating group did not show any difference between the groups, and the expression of OPN (Osteopontin) was high in the Control, Mn 10,000, and Mn 20,000 groups. RUNX2 (a key transcription factor for osteoblast differentiation) was expressed most highly in the mPEG-PLGA (Mn 30,000) coating group. p-ERK was very strongly expressed in mPEG-PLGA Mn 20,000, Mn 30,000, and Untreated Mg. In particular, it was highest in the Untreated Mg group. This suggests that the intracellular signaling pathway is strongly activated in both the polymer coating (especially the high-molecular-weight condition) and untreated Mg. β-Actin is stably expressed in all samples and is used as a quantitative standard.

Figure 7B compares the expression of bone-related proteins and signaling activity levels in mPEG-PLGA-coated Mg samples with various dexamethasone concentrations. While BMP-2 levels were high in the Dex 3 and 1.5 mg/mL concentration groups, BMP-7 and OPN expression tended to be similarly low in all Dex 7.5, 4.5, 3, and 1.5 mg/mL concentration groups. Untreated Mg also showed extremely low expression of osteogenic factors. The expression of the osteogenic transcription factor RUNX2 was high in all dexamethasone-treated groups, particularly in Dex 4.5 and 3 mg/mL and untreated Mg. The Dex-treated group also showed a significant increase in p-ERK compared to the control group, demonstrating that Mg corrosion and drug stimulation strongly induce Extracellular Signal Regulated Kinase (ERK) (signaling pathway activation) signaling. Taken together, these results indicate that the combination of Mg corrosion products and Dex strongly activates intracellular signaling through the ERK pathway.

**Figure 8:**
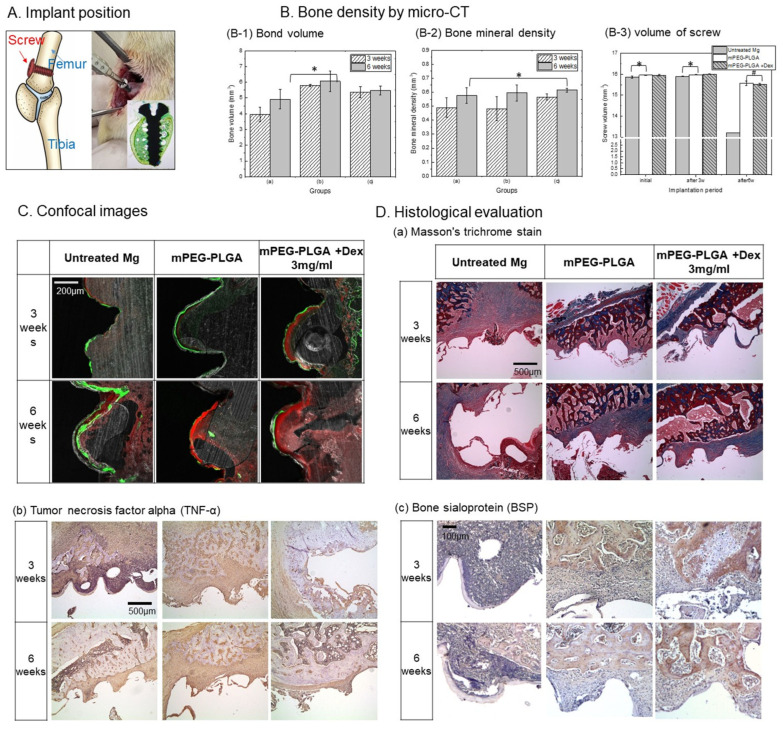
Rat femur implantation and peri-implant bone regeneration. (**A**) Schematic of screw placement in the distal femur. (**B**) Micro-CT: (**B-1**) peri-implant bone volume and (**B-2**) BMD at 3 and 6 weeks for (**a**) untreated Mg, (**b**) mPEG-PLGA, (**c**) mPEG-PLGA + Dex 3 mg/mL, (**B-3**) Volume of implant screw measured by micro-CT at initial placement (**C**) Confocal images of the bone–implant interface. (**D**) Histology: (**a**) Masson’s trichrome, (**b**) TNF-α, (**c**) BSP at 3 and 6 weeks.

Figure 8 shows the effects of processing a magnesium implant with a pH-responsive mPEG-PLGA coating and Dex on inflammatory responses and bone formation in a rat femoral implant model as shown in Figure 8A. Quantitative analysis of bone volume and optical density (Figure 8B) revealed that bone volume was improved in the treated groups compared to the untreated group, with the mPEG-PLGA group (b) showing the highest bone volume. However, bone optical density (B-2) was highest in the dexamethasone-treated group at both 3 and 6 weeks, demonstrating improved bone quality. In implant volume degradation over 6 weeks (Figure 8B(B-3)), the untreated Mg group showed minimal change in screw volume at 3 weeks (approximately 15.90 ± 0.008 mm^3^), despite observable surface corrosion in histological sections. This apparent volume preservation at 3 weeks reflects the accumulation of magnesium corrosion products (primarily Mg(OH)_2_ and apatite deposits) on the implant surface (Figure 3B,C), which compensate for the underlying metal loss and maintain the overall volumetric profile. However, at 6 weeks, the untreated Mg group exhibited a significant volume reduction to approximately 13.196 mm^3^, representing a loss of approximately 18–20% of the initial screw volume, indicating progressive material degradation despite the protective corrosion product layer. The mPEG-PLGA groups maintained stable screw volume at both 3 and 6 weeks, with negligible volume loss (approximately 0.5–1.0% over 6 weeks), demonstrating effective corrosion inhibition by the polymer coating.

Confocal image analysis (Figure 8C) confirmed that the mPEG-PLGA + Dex group exhibited thick, continuous bone formation at the bone–implant interface at both 3 and 6 weeks. Untreated magnesium exhibited a discontinuous interface and minimal bone neogenesis, whereas the Dex-treated group exhibited superior biocompatibility and tissue integration. Figure 8D(a) shows Masson’s trichrome staining of the tissue around the implant. Blue mainly represents collagen/fibrous tissue and immature bone matrix, and red represents cytoplasm/bone tissue. In the untreated group, a large cavity was formed along the implant interface, and thin fibrous tissue and inflammatory cells (red and black nuclei) were observed. In the mPEG-PLGA group, a thin blue layer was formed at the implant site, and the bone structure was relatively well maintained without a large cavity at 6 weeks. In the dexamethasone-added group, the densest and organized blue layer was observed at the implant interface, and stabilized collagen fibrous tissue (blue) could be confirmed at 6 weeks. Immunohistochemical staining to confirm the expression of tumor necrosis factor (Figure 8D(b)) showed that in the untreated group, the strongest brown expression was observed at the implant interface and throughout the surrounding tissue. In the mPEG-PLGA group, expression was mainly limited to the interface between bone and fibrous tissue. The dexamethasone-added group shows the weakest brown expression among the three groups at 3 weeks, but at 6 weeks, brown expression increases noticeably, especially around the bone and in tissues that appear to be newly formed. Bone Sialoprotein (BSP) immunohistochemical staining results (Figure 8D(c)): Secreted by osteoblasts, it is particularly important for determining the mineralization of new bone. In the untreated group, tissue around the implant was densely filled with dark purple nuclear staining, with almost no detectable brown BSP signal. In the mPEG-PLGA + Dex group at 3 weeks, the strongest BSP expression among the three groups is observed on the bone surface and in the newly formed bone matrix. At 6 weeks, both coating groups maintain well-maintained bone structure, and the new bone matrix is abundantly stained brown. These findings indicate that the most stable and continuous bone formation occurred in the coated groups, particularly with dexamethasone.

## 4. Discussion

This study aimed to develop a pH-responsive mPEG-PLGA coating to overcome the rapid corrosion and alkalinization of magnesium implants, thereby promoting bone integration, and to evaluate its in vivo performance when loaded with dexamethasone. Existing magnesium implants have been limited in clinical application due to rapid corrosion and hydrogen production upon exposure to body fluids, as well as the resulting adverse effects (gas pockets, tissue necrosis, and inflammation due to elevated pH). This study introduced a design in which the coating’s degradation selectively occurs in a magnesium corrosive environment (selective dissolution depending on the surrounding pH), thereby initially protecting the magnesium. When exposed to alkalinity due to localized damage to the material, the polymer rapidly dissolves, thereby alleviating the local alkalinization environment. The PLGA selected in this study is composed of a copolymer of polylactic acid and polyglycolic acid linked by ester bonds, and is hydrolyzed into lactic acid and glycolic acid, destroying the structure of PLGA and increasing its solubility [20]. Under alkaline conditions, ester bond hydrolysis of PLGA proceeds more rapidly than in neutral or acidic environments. This reaction mechanism involves nucleophilic attack of hydroxide ions on the carbonyl carbon of the ester bond, forming carboxylate and alcohol groups. This process breaks down the polymer chain into smaller, water-soluble fragments. To examine the pH-selective solubility of mPEG-PLGA, spheres were formed in polyvinyl alcohol (PVA). At pH 10, the sphere surface collapsed after 1 day, and rapid dissolution occurred after 2–3 days. The UV-Vis spectrum shows a typical absorbance curve at pH 7 (neutral), as the polymer spheres are barely soluble and there are few degradation products. Comparatively, at pH 10, many polymer degradation products are released into the liquid phase. During degradation at pH 10, the rapid breakdown of the coating generated numerous suspended polymer fragments, causing particle-induced light scattering to dominate over true molecular absorption. This intense scattering distorted the UV-Vis baseline downward, resulting in an apparent negative signal. Such optical phenomena are characteristic of highly scattering systems, as previously documented where turbidity interferes with the standard absorption-peak height relationship [21]. When spheres dissociate under alkaline conditions, the resulting polymer fragments become finely dispersed in the solution and remain suspended, which enhances light scattering in the low-wavelength (~330 nm) region. Around 430 nm, absorption can be observed from small-molecule degradation products generated as polymer particles break down into smaller particles or from specific bonds in the polymer (e.g., ester carbonyl, conjugated C=O). The 560 nm region is interpreted as a characteristic peak resulting from light scattering by products generated during dissolution.

Before utilizing these properties, it is important to select a PLGA with an appropriate molecular weight to achieve selective biodegradability. The lower the molecular weight of PLGA, the lower the mass of each unit, weakening intermolecular bonds and accelerating the degradation rate. As the molecular weight of PLGA increases, the intensity of the C=O stretching vibration (near 1700 cm^−1^) decreases due to the decrease in the relative concentration of carbonyl groups relative to the total molecular weight [22]. In this study, a copolymerization method with monomethoxy polyethylene glycol (mPEG) was applied to improve the hydrophilicity and degradability of PLGA. Compared to PLGA, mPEG has a longer chain structure, which increases intermolecular entanglement, and this limits the vibrational freedom of the carbonyl group due to the bonding of high-molecular-weight PLGA and mPEG (Figure 2C). The –CH_2_–CH_2_–O– structure of PEG increases with the bonding with high-molecular-weight PLGA, increasing the number of hydrogen-bondable oxygen atoms and enhancing hydrophilicity [23]. These properties mitigate insolubility and aggregation issues in biological environments and enhance the durability of the coating [24].

The coating layer (Figure 2C) showed a reduced peak intensity of C–H stretching vibrations at 3000–2800 cm^−1^ compared with the polymer spheres (Figure 2B), which can be attributed to the altered molecular packing of mPEG-PLGA on the Mg surface. This is attributed to the relatively flat arrangement of mPEG-PLGA on the Mg surface, which restricts vibrational degrees of freedom and reduces C–H stretching intensity, consistent with increased intrachain interactions [25]. In addition, the overall strong C–O peak (1100 cm^−1^) on the coated surface is due to the surface structure being modified during the coating process. Mn 10,000 has an incomplete coating and agglomerated particles due to its low molecular weight, resulting in an uneven and rough surface. Mn 20,000 formed a uniform surface with a regular, wave-like microstructure, whereas Mn 30,000, owing to its higher molecular weight and viscosity, produced a smoother surface but with potentially reduced coating efficiency [26]. The Mn 20,000 mPEG-PLGA group is stable in the C–O region and has high hydrophilicity, which promotes cell adhesion and is a favorable condition for biocompatibility, and the regular surface pattern will have a uniform drug-release ability.

This is consistent with the surface characteristics shown in surface roughness (Rq), contact angle, wetting energy, and work of adhesion. Mn 20,000 exhibited the lowest contact angle (39.78°) and highest wetting energy (55.97 mN/m), indicating the most hydrophilic surface. In general, increased surface roughness facilitates spreading of water droplets and can enhance the apparent hydrophilicity. However, incorporation of Dex increased surface roughness and drug loading while introducing a trade-off in the form of higher hydrophobicity. Increased surface roughness and hydrophilicity can maximize interactions with biological fluids, proteins, and cells across the entire implant surface. Surface hydrophilicity has a positive effect on cell adhesion, proliferation, and tissue integration. In the Dex-loaded Mn 20,000 group, roughness remained high but the contact angle increased to a level comparable to the Mn 10,000 group. The bulk SBF pH measurements’ behavior may be explained by partial surface coverage by Dex, which likely reduces the effective exposure of hydrophilic groups and thereby alters drug-release behavior and interfacial reactions in biological environments [27]. The change in hydrophobicity due to the addition of Dex can be applied to control the inflammatory environment or drug release timing, and these findings suggest that fine-tuning of surface wettability and adhesion is critical for precisely controlling biological responses.

Untreated Mg showed uneven micro-precipitation and localized corrosion after SBF immersion, and the precipitate layer was thin and the bonding strength was weak. This demonstrates the limitations of Mg implants, which suffer from rapid corrosion and surface instability in the body environment. The magnesium-coated group (Mn 10,000–30,000) maintained a relatively low pH of the solution during SBF immersion compared to the untreated Mg(a) group. This is because the polymer coating layer formed on the magnesium surface effectively inhibits direct corrosion of the magnesium metal. Even if the coating film is partially dissolved or damaged over time, the initial corrosion inhibition effect alone shows a significantly gentler pH increase curve compared to the untreated group. In addition, since the degradation products of PLGA-based polymers are decomposed into organic acids such as lactic acid and glycolic acid, they have the effect of lowering the alkaline pH of the acidified surrounding body fluid [12]. The thickness and durability of the coating layer are excellently maintained in the FIB cross-section, suggesting that the coating and drug incorporation provide both mechanical protection and chemical stability. The decrease in Ca concentration in SBF can be explained by rapid Mg dissolution, whereby released Mg^2+^ and OH^−^ react with Ca and P in solution and precipitate as a calcium phosphate/apatite layer on the Mg surface. Accordingly, Mg^2+^ concentration in the medium increases markedly as corrosion resistance decreases. An initially unstable Mg(OH)_2_ corrosion layer is formed on the surface, which is destroyed again by Cl^−^, high pH, tensile stress, etc., and local corrosion is repeated [28]. In the same context, the untreated group steadily lost weight over the course of 7 days, finally reaching approximately 82%. This indicates that the sample was dissolved and lost due to strong corrosion. In the coated groups (Mn 10,000–30,000), sample weight increased slightly on days 2 and 3, likely due to surface growth of oxides or precipitates, and remained within 97–112% of the initial mass by day 7. In particular, the Mn 30,000 coating group showed little or no weight loss or even an increase, demonstrating a very stable protective effect [3]. Untreated Mg exhibited a typical pattern of rapid initial corrosion followed by a gradual slowdown, while the coated group exhibited significantly suppressed initial corrosion and only moderate mass changes over the long term. This suggests that the polymer coating and self-passivation by corrosion products act simultaneously to protect the metal substrate over the long term. Subsequent deposition of corrosion products (self-passivation) acts as a barrier to further degradation, and in metallic systems, repeated cycles of film breakdown and re-passivation typically continue until most of the protective layer is consumed [29]. Although the bulk SBF pH measurements (Figure 4A) indicated an average pH of 9.2–9.5 around mPEG-PLGA-coated Mg, the local pH immediately adjacent to actively corroding sites is expected to be substantially higher due to continuous generation of OH^−^ and Mg(OH)_2_, likely exceeding pH 10 in microdomains. While our microsphere degradation experiments (Figure 1B,C) and coating morphology changes (Figure 3B,C) provide indirect evidence that the coating responds selectively to these alkaline microenvironments. Future work employing spatially resolved pH mapping at the coating–Mg interface will be valuable to confirm that local pH elevations at corrosion sites indeed reach the threshold (≥pH 10) required for accelerated mPEG-PLGA degradation, thereby more definitively distinguishing pH-responsive dissolution from simple bulk hydrolysis.

In this study, a pH-responsive mPEG-PLGA polymer coating and dexamethasone sustained-release system were applied to address the problem of tissue inflammatory response, and long-term drug release characteristics, ion release kinetics, and biological immune response inhibition effects were comprehensively evaluated. To ensure experimental reproducibility across in vitro release tests and in vivo specimen fabrication, Dex concentrations were standardized based on the preparation volume (mg/mL). While the absolute mass of Dex incorporated into the solid coating layer remains difficult to quantify directly, dissolution testing confirmed a functional cumulative release. For example, the 3 mg/mL Dex group maintained a final cumulative concentration of approximately 0.025 mg/mL over three weeks.

In general, long-term use of systemic corticosteroids has been shown to cause osteoporosis due to their ability to inhibit the proliferation of osteogenic precursor cells, a potentially serious side effect [30]. However, according to the literature, continuous exposure to Dex in the range of approximately 63 ng/mL or less is reported to be beneficial in promoting osteogenic differentiation of MSCs [31]. The dexamethasone treatment groups exhibited an initial burst release (rapid release of drug from the surface), followed by a gradual slowdown and a plateau in release. This pattern is typical of high-concentration coatings. The cumulative Dex release over three weeks was 0.018–0.028 mg/mL, corresponding to an average release rate of approximately 35.71–55.56 ng/mL per hour. The dose was selected to ensure long-term safety within the range of 63 ng/mL or less. The 3 mg/mL group initially exhibited a lower release, but over time, it exhibited sustained and stable release (diffusion and polymer degradation). The optimized distribution of the drug or polymer within the coating structure resulted in a uniform release over a long period of time. When the drug loading was well matched to the polymer matrix, diffusion-controlled release could be sustained over prolonged periods [32]. However, the initial burst release observed in the first 2 days (Figure 5A) warrants careful consideration in the context of clinical translation. While our rat femur model showed no overt systemic adverse effects and histology at 3 and 6 weeks indicated normal bone formation and reduced TNF-α expression (Figure 8D), the small implant size and limited drug load in rodents may not fully reflect the scenario of large orthopedic implants in humans. Moreover, large-animal studies with clinically relevant implant dimensions and mechanical loading will be essential to confirm that the burst-release phase does not compromise early healing or increase infection susceptibility.

The observed TNF-α and IL-1β secretion profiles in RAW 264.7 macrophages closely reflect the effects of Mg corrosion, surface coating, and Dex release on the local inflammatory response. LPS, a component of the cell wall of Gram-negative bacteria, is a common inflammatory inducer that strongly activates macrophages through the TLR4 receptor. Exposure of LPS-activated RAW 264.7 macrophages for 48 h activates NF-κB and induces TNFα and IL-1β gene expression, a sufficient period for protein production and secretion, providing a sufficient time window for robust detection of secreted cytokines by ELISA [33]. Untreated Mg strongly stimulates macrophages to secrete high concentrations of TNFα and IL-1β due to rapid corrosion and subsequent pH increase, Mg^2+^/OH^−^ accumulation, and reactive oxygen species (ROS) production [34], which is consistent with previous reports showing that rapid corrosion of magnesium alloys induces innate immune system activation and inflammatory cytokine secretion.

Dex coating (d–g) appeared to effectively suppress the inflammatory phenotype of macrophages through locally released Dex inhibition of NF-κB and MAPK signaling, and reduction in COX-2 and iNOS expression [31]. In particular, the Dex 7.5 and 4.5 mg/mL coating groups showed significant decreases in both TNF-α and IL-1β, maintaining an anti-inflammatory state close to the negative control group despite stimulation by magnesium corrosion. This can be interpreted as a result of a synergistic effect of a dual mechanism in which the polymer coating primarily controls excessive corrosion of Mg, and the simultaneously released Dex secondarily suppresses the production of inflammatory cytokines. As the initial release concentration is likely to approach the cytotoxic range at the highest Dex concentration, it is important to set the optimal concentration together with the results of cell experiments such as WST 8 and ALP. In the WST 8 results using osteoblast MC3T3-E1 cells, the Mn 20,000 and Mn 30,000 coatings were interpreted to have acted as stable substrates even in a cell culture environment, as they formed a dense Ca–P precipitate layer and had excellent corrosion inhibition effects, as observed in SBF deposition and EDX analysis. However, high-concentration coatings such as Dex 7.5 mg/mL showed a slowing down of cell proliferation increase in long-term culture (4 days), suggesting that continuous exposure to high-concentration Dex may have a partial inhibitory effect on osteoblast proliferation. The fact that the oxidative stress in the Dex-coated group significantly decreased over time in the H_2_O_2_ analysis can be seen as a result of the combined effects of Dex’s inherent anti-inflammatory and antioxidant effects and the reduction in ROS production due to corrosion mitigation. This is consistent with the previous result that TNFα and IL-1β secretion was decreased in a Dex concentration-dependent manner in RAW 264.7, and supports that mPEG PLGA/Dex coating contributes to reducing inflammation and oxidative stress in both innate immune cells and osteoblasts. Meanwhile, the increase in ALP activity was most pronounced in the Dex 3–4.5 mg/mL coating group, indicating that this concentration range promotes early osteoblast differentiation. Conversely, TRACP activity was highest in untreated Mg, and excessive loading such as Dex 7.5 mg/mL may negatively affect long-term cell proliferation and differentiation even though the initial anti-inflammatory effect is strong. Thus, Dex concentrations at or below 4.5 mg/mL (groups f, g) showed a clear inhibitory effect on TRACP activity, which is likely beneficial for bone formation by reducing osteoclast activity and bone resorption.

BMP 2/BMP 7 heterodimer is one of the most potent osteoinductive factors known, playing a key role in inducing osteogenic differentiation of MSCs and promoting bone formation [35]. In the mPEG PLGA coating group (Mn 10,000–30,000), BMP 2 and BMP 7 expression was maintained at the control level, indicating that the polymer coating provides a microenvironment favorable for osteogenic differentiation by alleviating corrosion toxicity and protecting the BMP axis. In particular, the high OPN levels in the Control, Mn 10,000, and Mn 20,000 groups suggest that these coatings create a surface environment more suitable for bone matrix formation and cell adhesion. RUNX2 is a master transcription factor for osteoblast differentiation and is well known to be an essential factor for both bone-specific gene expression and chondrocyte hypertrophy [36]. The highest expression of RUNX2 in the Mn 30,000 coating group can be interpreted as a result of the high-molecular-weight coating enabling MSCs to actively activate the osteogenic differentiation program through surface stabilization and corrosion mitigation. The high RUNX2 and p-ERK expression in untreated Mg (Figure 7A) reflects the intrinsic osteogenic potential of magnesium ions, well documented to promote osteogenic differentiation. However, this osteogenic signal was not translated into effective bone matrix deposition or in vivo bone formation (Figure 8).

p-ERK is the phosphorylated, active form of ERK and plays a crucial role in various intracellular signaling pathways. ERK activity is required for BMP 2-induced RUNX2 acetylation and stabilization, and can contribute to osteogenesis by promoting the expression of osteogenic genes and cell growth and differentiation in various cells [37].

However, p-ERK can also be involved in osteoclast differentiation or activation depending on the situation, so excessive activation can act toward promoting bone resorption [38]. In this study, the strong activation of p-ERK in Mn 20,000, Mn 30,000, and untreated Mg indicates that the stress and signaling stimulation induced by Mg corrosion are very strong. In the coated group, this activation is likely to contribute to bone formation in conjunction with the BMP 2/BMP 7–RUNX2 axis, but in untreated Mg where BMP 2/7 and OPN are inhibited, p-ERK has a duality that can induce bone destruction or abnormal differentiation. In this study, the fact that BMP 2 was relatively well maintained in Dex 3 and 1.5 mg/mL, and RUNX2 expression was high in all Dex groups suggests that Dex can amplify osteogenic differentiation signals through the BMP 2/RUNX2 axis. However, the overall low levels of BMP-7 and OPN at all Dex concentrations suggest that Dex may negatively affect bone matrix formation and BMP-7-mediated signaling while maintaining BMP-2-dependent RUNX2 activity. The pattern of simultaneous high activation of RUNX2 and p-ERK at 3–4.5 mg/mL of Dex suggests strong stimulation of osteogenic differentiation signals, but the increase in RUNX2/p-ERK in the presence of BMP-2/7 and OPN inhibition, as in untreated Mg, is likely to be stress-induced or an abnormal response, and therefore requires careful, context-dependent interpretation with respect to Dex concentration and microenvironment.

In a rat femur implantation model, the bone volume around the implant was increased in all surface-treated groups compared to untreated Mg, and the mPEG PLGA group (b) showed the highest bone volume. This suggests that the polymer coating suppressed rapid corrosion of Mg and maintained mechanical stability, thereby providing a favorable environment for preserving surrounding bone structure and increasing bone mass [39]. The mPEG-PLGA-coated groups maintained volumetric stability throughout the 6-week period, with minimal volume loss (~0.5–1.5%). This >10-fold reduction in volume loss compared to untreated Mg (18–20% loss) directly demonstrates that the polymer coating suppresses the initiation and propagation of localized corrosion pits by maintaining a stable physical barrier and by buffering alkaline pH conditions through its degradation. The nearly identical volume retention between the unloaded mPEG-PLGA group (b) and the drug-loaded mPEG-PLGA + Dex group (c) indicates that dexamethasone loading does not compromise the anti-corrosion efficacy of the polymer matrix, an important safety consideration for clinical translation.

Meanwhile, the bone mineral density (BMD) was highest in the mPEG PLGA-coated group (c) containing Dex at both 3 and 6 weeks, indicating that the sustained release of Dex contributes to the improvement of bone quality (degree of mineralization) beyond simple bone mass increase. This is consistent with the results observed in vitro in which increased ALP activity, BSP expression, and BMP 2/RUNX2 pathway activation were observed in the Dex-coated group. This trend was also evident in bone–implant interface analysis using confocal images. In untreated Mg, new bone formed around the implants thinly and discontinuously at both 3 and 6 weeks, with little bone–implant contact in some areas. This is interpreted as being due to local alkalinization, hydrogen bubbles, and tissue necrosis caused by corrosion, which hindered osseointegration [40]. In particular, the formation of a thick, continuous band of new bone around the implant in the mPEG PLGA + Dex group can be seen as direct evidence that the coating promoted osteoblast activity and bone matrix accumulation beyond simple corrosion blocking. In Masson’s trichrome staining, the Dex-added group showed the thickest and most organized blue layer at the implant border, and at 6 weeks, stabilized collagen fibers and dense bone matrix were formed, suggesting the most favorable condition for long-term bone regeneration and bone–implant bonding. This is consistent with the pattern of increased ALP, decreased TRAP, and increased BMP 2/RUNX2 expression in the Dex 3–4.5 mg/mL coating group in vitro. A similar trend to the in vitro RAW 264.7 results was observed in TNFα immunohistochemical staining, which reflects an inflammatory response. The Dex-added group showed the weakest TNFα expression among the three groups at 3 weeks, suggesting that it effectively suppresses the early inflammatory response. At 6 weeks, some brown signals increased, but these were mainly observed around new bone or in tissues where remodeling processes were occurring, suggesting that this is likely due to the expression of more remodeling-related cytokines, suggesting that these signals are more likely associated with remodeling-related cytokine expression rather than persistent acute inflammation. This indicates that while initial excessive inflammation is suppressed by sustained release of Dex, an appropriate level of immune and inflammatory response necessary for long-term bone remodeling is maintained [41]. BSP is an early bone matrix protein secreted by osteoblasts and is particularly used as an indicator of new bone mineralization. At 6 weeks, abundant brown signals were observed in both mPEG-PLGA and mPEG-PLGA + Dex coating groups, confirming that stable new bone formation and mineralization were in progress. This corresponds well to the increased ALP activity in the Dex coating group in vitro and the increased RUNX2, BMP 2, and BSP family proteins on Western blot. In summary, the pH-responsive mPEG-PLGA coating suppresses rapid corrosion of Mg implants and the resulting toxicity and inflammation, providing a favorable environment for maintaining bone mass and structure. When Dex groups at an appropriate concentration (3–4.5 mg/mL in this study) are combined here, they can effectively reduce early inflammation (TNFα) while promoting bone formation and mineralization through the BMP 2/RUNX2 axis and BSP expression, thereby achieving the best BMD and bone–implant bonding in the long term [42]. This is consistent with the results of alleviating the pH/ionic environment, reducing ROS, suppressing macrophage inflammation, and promoting osteoblast differentiation observed in vitro, and supports the importance of a coating strategy that achieves not only simple corrosion inhibition but also local inflammation control + promotion of bone regeneration at the same time in the clinical application of biodegradable Mg implants.

## 5. Conclusions

In this study, we developed a pH-responsive mPEG-PLGA coating containing dexamethasone as an integrated corrosion-control and immunomodulatory system for biodegradable magnesium implants. By linking degradation behavior, drug release, cellular responses, and in vivo bone regeneration in a rat femur model, we show that this coating not only attenuates corrosive and inflammatory effects of magnesium but also establishes a stable osteogenic peri-implant microenvironment. The mPEG-PLGA coating homogenized magnesium dissolution, mitigated alkaline shifts, and reduced Mg^2+^ release in vitro, improving MSC and osteoblast viability and reducing oxidative stress. pH-responsive Dex release dose-dependently suppressed TNF-α and IL-1β in macrophages while upregulating ALP, BSP, and the BMP-2/BMP-7–RUNX2 axis, and Western blot analysis indicated that Dex coatings enhanced RUNX2 and p-ERK without shifting toward a stress- or resorption-dominant pathway. Consistent with these mechanisms, coated implants significantly enhanced peri-implant bone formation in vivo: micro-CT showed increased bone volume with mPEG-PLGA and higher bone density with Dex-containing coatings at 3 and 6 weeks, while confocal and histological analyses revealed thicker, more continuous bone–implant contact, fewer cavities, preserved cancellous architecture, reduced early TNF-α staining, and sustained BSP-positive mineralization of interfacial new bone. Overall, the pH-responsive mPEG-PLGA/Dex system transforms a corrosive, inflammatory niche into a regenerative microenvironment that couples immunomodulation with osteogenesis. However, because this work uses a single steroid (dexamethasone) and relatively simple cell and normal rat models, further studies in large animals, disease models, and multi-drug systems are required to define long-term safety and optimal therapeutic windows.

## Data Availability

The original contributions presented in this study are included in the article/Supplementary Material. Further inquiries can be directed to the corresponding author(s).

## Supplementary Materials

The following supporting information can be downloaded at https://www.mdpi.com/article/10.3390/polym18020303/s1, Figure S1. UV–Vis drug release curves for (a) standard solution and samples measured at (b) 6 h, (c) 1 day, (d) 7 days, and (e) 21 days (Extended information of Figure 5A).
