# Supplementary material for: pH-Responsive mPEG-PLGA/Dexamethasone Coatings for Corrosion Control and Osteo-Immune Modulation of Biodegradable Magnesium
Source: Polymers (Basel). 2026 Jan 22;18(2):303. doi: 10.3390/polym18020303 (PMC12846112; doi:10.3390/polym18020303)
Supplement: Supplementary file 1 [file polymers-18-00303-s001.zip › polymers-4081823-supplementary.pdf]

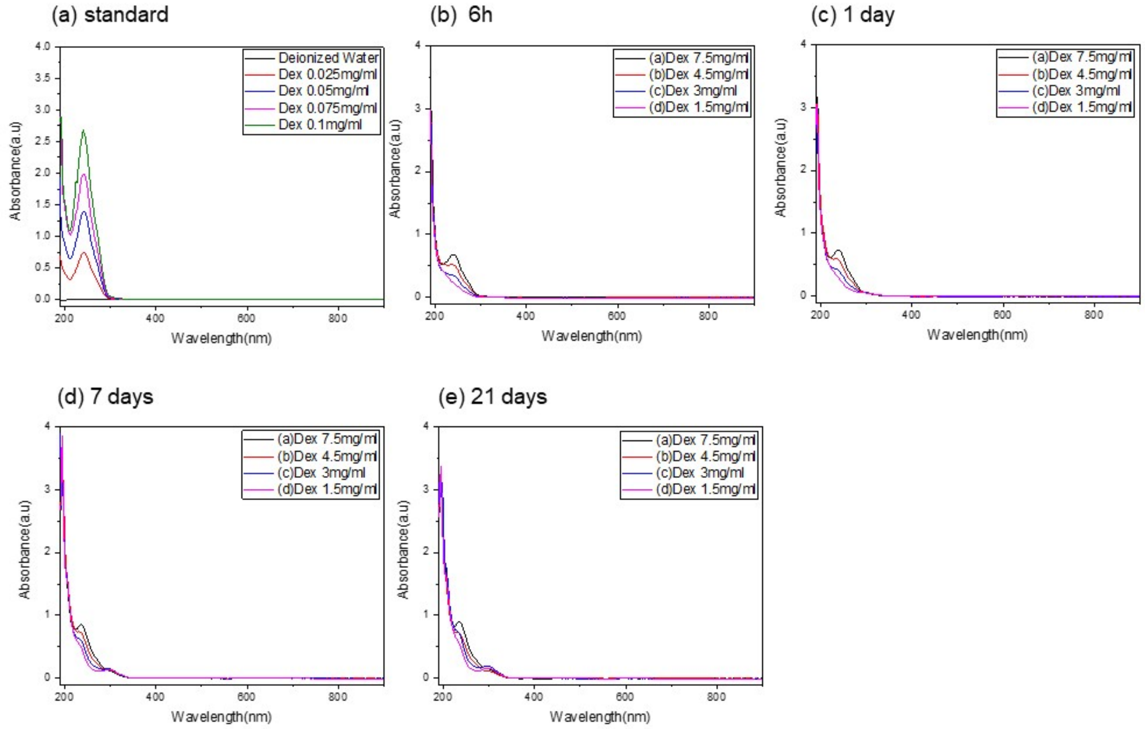

Figure S1. UV–Vis drug release curves for (a) standard solution and samples measured at (b) 6 h, (c) 1 day, (d) 7 days, and (e) 21 days (Formerly Figure 5A).
